# The Rising Challenge of Poor Health Literacy of Patients with Systemic Sclerosis: Preliminary Data Identify Important Unmet Needs in an Italian Cohort

**DOI:** 10.3390/nursrep14010043

**Published:** 2024-03-05

**Authors:** Khadija El Aoufy, Maria Ramona Melis, Paolo Iovino, Stefano Bambi, Chiara Lorini, Guglielmo Bonaccorsi, Ilaria Galetti, Carla Garbagnati, Paola Canziani, Silvia Tonolo, Marco Mitola, Serena Guiducci, Daniel E. Furst, Marco Matucci-Cerinic, Laura Rasero, Silvia Bellando-Randone

**Affiliations:** 1 Department of Health Science, University of Florence, 50134 Florence, Italy; paolo.iovino@unifi.it (P.I.); stefano.bambi@unifi.it (S.B.); l.rasero@unifi.it (L.R.); 2 Department of Experimental and Clinical Medicine, University of Florence, 50134 Florence, Italy; ramona.melis@ymail.com; 3 Health Literacy Laboratory, Department of Health Science, University of Florence, 50134 Florence, Italy; chiara.lorini@unifi.it (C.L.); guglielmo.bonaccorsi@unifi.it (G.B.); 4 GILS (Gruppo Italiano Lotta alla Sclerodermia), 50134 Milan, Italy; ila.galetti@gmail.com (I.G.); pcanziani@italex.it (P.C.); 5 FESCA (Federation of European Scleroderma Associations), 7500 Saint Maur, Belgium; 6 ANMAR Onlus (Associazione Nazionale Malati Reumatici), 00145 Rome, Italy; 7 ASSMAF (Associazione per lo Studio della Sclerosi Sistemica e delle Malattie Fibrosanti), 50143 Florence, Italy; 8 Rheumatology Unit, Department of Experimental and Clinical Medicine, Section of Internal Medicine, Azienda Ospedaliero-Universitaria Careggi (AOUC), University of Florence, 50134 Florence, Italy; serena.guiducci@unifi.it (S.G.); dan@furst.us.com (D.E.F.); silvia.bellandorandone@unifi.it (S.B.-R.); 9 Division of Rheumatology, David Geffen School of Medicine, University of California, Los Angeles, CA 90095, USA; 10 Unit of Immunology, Rheumatology, Allergy and Rare Diseases (UnIRAR), IRCCS San Raffaele Hospital, 20132 Milan, Italy; matuccicerinic.marco@hsr.it

**Keywords:** health literacy, systemic sclerosis, scleroderma, observational study, self-care

## Abstract

Rationale and aim: Health literacy (HL) is pivotal for the successful self-management of chronic diseases. Little HL information is currently available in SSc patients; therefore, the present study aims at evaluating the HL levels in an Italian cohort of SSc patients. Methods: SSc patients were enrolled with the support of Italian patient associations, from September 2022 to March 2023. Health literacy characteristics were derived from the Health Literacy Scale European Questionnaire-16 (HLS-EU-Q16), consisting of 16 items designed on a four-point Likert scale ranging from “very difficult” to “very easy”, and three HL levels were identified: inadequate HL (0–8 score); problematic HL (9–12 score); and sufficient HL (13–16 score). Results: Enrolled patients (n = 57, mean age = 59 years, SD = 13.2) were mostly female (98.2%), partnered (73.7%), and unemployed or retired (67.9%). Almost half of SSc patients were diagnosed more than 10 years ago, with first symptoms appearing on average 19 years ago (SD 10.5). In 63% of the participants, the overall health literacy skills were inadequate, or problematic, especially in the health care and disease prevention domains. Indeed, 49.2% of the patients declared difficulty in finding information on treatments for illnesses and where to get professional help (42.1%), 47.6% found difficulty in retrieving information on how to manage mental health problems, and 40.4% declared difficulties in judging whether the information on health risks in the media was reliable. Conclusions: Our findings show that SSc patients have inadequate or problematic levels of HL, suggesting the need for periodic screenings to uncover poor health literacy skills and to provide tailored and understandable educational material. This study was not registered.

## 1. Introduction

Systemic sclerosis (SSc) is an immune-mediated rheumatic disease characterized by vasculopathy, disimmunity, and significant fibrosis of the skin and internal organs [1]. The causes and processes of disease progression are still not fully understood, despite research improvements in an understanding of the disease over the last decades [1]. Thus, SSc remains a chronic, complex, and debilitating disorder, resulting in significant morbidity and mortality worldwide, a high morbidity burden, and a heavy impact on patients’ health-related quality of life (HRQoL) [2]. Recently, innovative drugs have been shown to reduce the fibrosis and complications of SSc, but complete disease remission remains an unmet need [3].

In practice, SSc patients must adhere to a variety of health care behaviors to self-manage their disease. For example, adherence to pharmacological therapy is of paramount importance, and substantial lifestyle and behavioral adjustments are key in everyday life. Moreover, learning to cope with the psychological and social implications is mandatory to help effectively manage the disease [4,5,6]. In fact, in SSc, adherence to self-care behaviors has led to better outcomes, including greater muscle strength, mobility, and health-related quality of life [7,8]. Unfortunately, there is evidence that self-care is lacking in this population [9]. 

In the last decades, increased attention has been devoted to self-care and its determinants, especially in the context of chronic conditions [10]. This research trend has led to the study of several additional health outcomes, including the health literacy construct (HL). According to the European Health Literacy Survey Project, HL represents a broad and inclusive concept, defined as the “knowledge, motivation and competencies to access, understand, appraise and apply health information in order to make judgments and take decisions in everyday life concerning health care, disease prevention and health promotion” [11]. 

Recent studies have underlined that, patients with chronic diseases and low levels of HL may develop adverse health outcomes, such as low treatment compliance, inadequate use of medical services, and poor quality of life [12,13,14]. 

Difficulties regarding HL are generally found in vulnerable groups, such as individuals with chronic disabilities and older adults [15,16], and patients with rheumatic diseases are at great risk of poor HL [17,18]. Current studies have shown that rheumatic patients have difficulties in understanding their condition and treatment options related to the disease, as well as problems in seeking support services. In rheumatic diseases, the findings underscore that HL may affect medication adherence, functional status, and patient activation [19]. This is worrisome because such outcomes, especially medication adherence, are associated with a higher mortality, the use of health care services, and a poorer quality of life [7,20]. 

Despite the current evidence, research on HL levels of rheumatic patients has mainly focused on systemic lupus erythematosus and rheumatoid arthritis [21,22], thus neglecting other vulnerable populations, such as SSc patients. 

As mentioned above, SSc is a rare and debilitating disease that has a chronic course, and for this reason, this population should undergo further examination regarding HL. To the best of our knowledge, only one Chinese study explored HL in SSc patients, finding that out of a sample of 428 patients, only 14% had adequate HL levels [23]. The scarcity of data on HL in SSc, especially in the European population, which is culturally diverse from China, prompted us to evaluate the HL levels in an Italian cohort of SSc patients.

## 2. Materials and Methods

### 2.1. Data Collection and Procedures

A descriptive observational study was performed on a sample of Italian SSc patients. Data were collected from September 2022 to March 2023, and SSc patients were enrolled through a convenience sample with the support of the ASSMAF (Associazione Sclerosi Sistemica e Malattie Fibrosanti, Florence, Italy), GILS (Gruppo Italiano Lotta alla Sclerodermia, Milano, Italy), and ANMAR (Associazione Nazionale Malati Reumatici, Rome, Italy) patient associations. 

Inclusion criteria were (a) an SSc diagnosis according to the 2013 ACR/EULAR criteria [24]; (b) ≥18 years old; and (c) a willingness to participate and informed consent. Exclusion criteria were (d) difficulties in understanding the Italian language and (e) visual or cognitive impairments. Before completing the data collection procedure, all patients signed an informed consent form. 

A data collection tool based on sociodemographic questions and the HL questionnaire, as specified in the instrument section, has been disseminated online through the patient associations that participated in the study. 

### 2.2. Instruments

The Italian version of the HLS-EU-Q16 (European Health Literacy Survey Questionnaire-16) was used to assess the HL skills of patients [25]. This scale consists of 16 items that measure the perceived ability to obtain, understand, evaluate, and use the health information for health promotion, disease prevention and health care, and maintain and promote health. The answers of the HLS-EU-Q16 are designed on a 4-point Likert scale, ranging from “very difficult” to “very easy”. To generate a score for the HLS-EU-Q16, answers are dichotomized into “fairly difficult” and “very difficult” (coded 0) and “fairly easy” and “very easy” (coded 1) categories. The total score of the HLS-EU-Q16 ranges from 0 to 16, with higher scores indicating greater HL levels. Three HL levels have subsequently been derived: inadequate HL (0–8); problematic HL (9–12); and sufficient HL (13–16). Respondents with more than 2 missing values in the HLS-EU-Q16 were excluded from the analysis [26]. According to a study conducted on the general population, the Italian version of the HLS-EU-Q16 has satisfactory validity and reliability [26]. In this study, the Cronbach’s Alpha for the total scale was also adequate at 0.87.

### 2.3. Statistical Analysis

The sociodemographic and clinical characteristics of the sample using means and standard deviations or median and interquartile ranges, as well as frequencies and percentages, were evaluated. We also reported absolute numbers, percentages, and means for each response category of the HLS-EU-Q16 items. The total score was split into three categories [e.g., inadequate (0–8), problematic (9–12), or adequate (13–16)] according to the instructions of the original author [26]. SPSS v.25^®^ was used for the descriptive analysis [27].

### 2.4. Ethical Considerations

This study was approved by the Institutional Review Board of the Regional Ethics Committee of the Tuscany Region, Italy (approval number: CE 20484), and designed in accordance with the principles of the Privacy Body of Law (Italian legislation numbers 196/2003 and 101/2018). Anonymity and data protection were assured with an individual sequential code number issued for each participant.

## 3. Results

Table 1 displays the clinical and sociodemographic characteristics of the 57 participants (mean age of 59 years, SD 13.2) that were almost exclusively female (98.2%), partnered (73.7%), unemployed or retired (67.9%), and had children (75.4%). Approximately, half of the patients had the SSc diagnosis more than 10 years ago, while the first symptom of disease manifestation (i.e., Raynaud phenomenon) appeared on average 19 years ago (Table 1).

Table 2 shows the HL levels of the sample in which approximately 63% of the sample exhibited either inadequate or problematic HL skills. The total score average of 10.61 (SD = 3.75) also confirmed problematic levels.

The descriptive analysis of the responses to the items of the HLS-EU-Q16 is shown in Table 3. 

Regarding the health care domain, 49.2% of the patients declared difficulty retrieving information on disease treatments and determining where to get professional help (42.1%). A smaller proportion (35.1%) also found it difficult to understand when to get a second opinion from another doctor. This (item #5) was also the one with the lowest average score (2.12, SD = 1.18).

Regarding the disease prevention domain, item #8 (“Find information on how to manage mental health problems like stress or depression”) had responses falling prevalently on the difficult categories of the scale (47.6%). Moreover, almost half of the patients declared difficulties judging whether the information on health risks in the media was reliable (40.4%). The lowest average score was found for item #8: “Find information on how to manage mental health problems like stress or depression” (2.09, SD = 1.12).

Regarding the disease promotion domain, the responses fell prevalently on the easy or fairly easy categories of the scale (56.1% to 79%). The lowest average score was found for item #14: “Understand advice on health from family members or friends” (2.23, SD = 1.17). 

## 4. Discussion

The aim of our study was to assess the HL levels in patients affected by SSc, a complex disease, and these patients need a significant awareness of their disease in order to optimize the use of health care resources and prevent disease progression. Our results highlighted that patients had a variety of difficulties retrieving relevant health care information to manage their disease. 

To the best of our knowledge, this is the first study describing HL levels in a cohort of Italian SSc patients. A significant proportion of our sample exhibited inadequate or problematic HL levels. Unfortunately, we do not currently have data on SSc populations with which to compare our findings, except the study conducted in China in which Zhuang et al. (2023) reported that 14.49% of patients had an adequate HL level, which is close to the HL level of the general Chinese population (14.18%) (reported in 2017) [23]. However, even though the prevalence in our sample is much higher in terms of HL inadequacy than that of the Chinese population, a comparison between the two populations is not possible, as the instruments used have different characteristics. Moreover, a cross-sectional, multi-center study of the World Health Organization (WHO) conducted in 17 countries of the European Union reveals significant variations in HL across countries, highlighting the necessity of tailoring the HL assessment to each specific country [28]. This study also adds that about 12% and 55% of Italian individuals have inadequate and problematic HL skills, respectively [28].

The results obtained in the HL health care domain underline that SSc patients complain about excessive difficulty in seeking health information. This is consistent with what is reported in the Chinese study, where the patients had difficulties understanding health information provided by the physician and following the instructions related to the prescribed drugs [23]. The difficulty described by our patients raises concerns about a possible poor treatment adherence in SSc patients, which is a key indicator of prognosis and disease progression across all chronic conditions [29,30]. This relationship is corroborated by the fact that HL is a powerful determinant of self-care behavior in chronic conditions [31]. The difficulty with these specific items may also reflect problems regarding the communication between patients and health care providers, which is a prerequisite to effective self-care behavior. It is important for health care providers to have ongoing effective communication with patients to uncover specific self-care needs and to promote adequate strategies to deal with them. Accordingly, several studies have shown that low HL levels negatively affect patients’ capacity to understand and access health information, communicate with health care providers, and recognize disease flares, thus resulting in poor health outcomes [32,33,34]. 

In the disease prevention domain, we noticed that patients struggled to understand the health warnings about correct behaviors and healthy lifestyles and recognize the importance of health screenings. These are important skills in the context of a chronic and complex disease such as SSc; therefore, patients should be educated through structured sessions to understand and interiorize these specific aspects and to change their lifestyles into healthier ones. Specifically, patients’ awareness and education should be focused on recognizing signs and symptoms typically related to common complications, such as interstitial lung disease, pulmonary arterial hypertension, and gastrointestinal problems, to allow clinicians to intervene promptly. 

Lastly, in the disease promotion domain, most SSc patients reported difficulty related to mental well-being or how to become healthier, particularly in judging which behavior is good or bad for their health and, consequently, for their disease. As reported by the WHO, disease promotion is the “process of enabling people to increase control over and to improve their health” and cannot be separated by the development of HL. 

Surprisingly, our results indicate that most of our patients found it easy to perform the skills related to the disease promotion domain. It is therefore presumed that these patients can self-manage their mental health, understand the information for adopting a healthy lifestyle, and distinguish behaviors that are important for their health from those that are not. 

However, it is also possible that more HL skills may not automatically translate to effective self-care behaviors. For example, well-educated people may have sufficient knowledge of how to deal with their health, but they do not perform important self-care practices, perhaps because of poor self-efficacy, poor social support, or other risk factors such as depression and low motivation [35]. Indeed, in their meta-analysis, Marciano et al. (2022) suggest a positive and significant association between HL and self-care [36], yet in chronic conditions the nature and the direction of this relationship is not clear [37]. This applies also to specific self-care behaviors, such as medication adherence, where Hyvert et al. (2023) confirm an unclear association [38]. Thus, these reflections warrant further studies that may enlighten how the relationship between HL and self-care works in SSc populations. 

Health outcomes are clearly influenced by self-care and self-management behaviors, among others. Self-management, which has been distinguished from self-care, more broadly delineates the healthy lifestyle behaviors adopted by all individuals for optimal growth and development or the preventive strategies performed to promote or maintain health [39,40]. Recent results of a meta-synthesis on self-management for chronic diseases identified three categories: focusing on illness needs, activating resources, and living with a chronic illness [41]. 

Moreover, the progression of the illness as well as the development of complications or comorbidities can significantly alter well-established self-management routines and overall adjustment. These may also apply to SSc patients and should be addressed by clinicians and health professionals. Thus, clarifying and discussing with the patients about when and who to communicate signs and symptoms to and how to access the health care system is mandatory. 

In a recent study on treatment options most preferred by SSc patients, the authors identified information that is central to the process of decision making, according to associated benefits and harms [42]. Their findings were in line with patient perceptions about essential outcomes of treatment in SSc and other rheumatic diseases, which showed that QoL and daily function were highly prioritized. To enhance these aspects and optimize the shared decision-making process, HL is required to be assessed and implemented [42]. Accordingly, another study focused on SSc patients’ preferences for the treatment of interstitial lung disease, highlighting the importance of shared decision-making processes in clinical practice. Namely, the results of qualitative and quantitative data showed that participants placed high importance on avoiding adverse effects and were willing and able to make trade-offs between attributes when considering treatment options; this suggests that risks of experiencing AEs can be balanced with symptom improvement or administration convenience [42]. This study has important implications for practice and research; however, further studies are required to confirm our findings, including comparisons with newly diagnosed patients and investigations of the predictors of HL in SSc patients. Specifically, we refer to the need for screening SSc patients for their HL levels and skills in order to provide materials and education tailored to meet their needs. Indeed, in these patients, low levels of HL should be addressed promptly to minimize poor health outcomes.

In clinical practice, it is important that patients develop optimal self-management skills, which entail the ability to monitor their disease and improve the use of cognitive, behavioral, and emotional strategies to maintain a satisfactory quality of life [43]. 

Patients with rare conditions, such as SSc, have more difficulties due to gaps in understanding their condition and treatment options, as well as a lack of support services [4]. Thus, the patient’s understanding of their disease is today an unmet need, which should be addressed by clinicians to foster patients’ self-management.

SSc patients may not be able to comprehend elements such as how and when to take prescribed medication, what signs and symptoms represent a disease flare, or when it is appropriate to visit the emergency department rather than a primary care physician. For these reasons, assessing HL represents a crucial factor to ease SSc patients’ access to medical care and enhance their knowledge of their condition. Also, to strengthen patient-centered health care, health professionals need to focus on the growth of the competence of the health system to satisfy complicated individual demands. In this regard, the evaluation of patients’ HL abilities is critical. Therefore, it is critical to have the appropriate skills to make adequate decisions and play an active role in the decision-making process, which might be facilitated by tailored patient education and understandable materials that are offered upon diagnosis.

Our study has some limitations, such as a small sample size and a descriptive nature of the study design, which did not allow us to make further inferential analyses and assess predictors of HL in our populations. However, the strength of the study is represented by the fact that no other studies assessing HL have been conducted previously in any European SSc population.

## 5. Conclusions

Based on our findings, it is important to reiterate that despite the fact that pharmacological treatment has led to very important therapeutic results and that experts have developed diagnostic criteria for the very early recognition of SSc, individuals do not necessarily self-manage optimally and vary in their ability to develop effective coping strategies. Indeed, assessing HL in SSc patients revealed inadequate/problematic levels of this specific health outcome and prompted us to further analyze it. Namely, we recommend that health care professionals involved in the care of SSc patients start considering HL as a part of periodic screenings to uncover poor health literacy skills in order to provide tailored and understandable educational materials to SSc patients, thus enhancing patient care to its fullest potential and ultimately enhancing patients’ quality of life. 

## Figures and Tables

**Table 1:** Sociodemographic and clinical characteristics of the participants (*n* = 57).

Gender (female), *n* (%)	56 (98.2)
Age (years), M (SD)	59.22 (13.2)
Age groups, *n* (%)	
>70 years	12 (21.1)
51–70 years	30 (52.6)
31–50 years	14 (24.6)
Civil status (married/partnered), *n* (%)	42 (73.7)
Occupation (unemployed/retired), *n* (%)	38 (67.9)
Education (<9 years), *n* (%)	20 (35.1)
Have children, *n* (%)	43 (75.4)
Years from diagnosis, *n* (%)	
<10	15 (26.3)
≥10	32 (56.1)
Time at symptom onset (years), M (SD)	19.0 (10.5)

Legend. M, mean; SD, standard deviation.

**Table 2:** Health literacy levels according to the 16-item European Health Literacy Survey Questionnaire.

HL level, *n*, %	
Inadequate (0–8)	17 (29.8)
Problematic (9–12)	19 (33.3)
Adequate (13–16)	21 (36.8)
Total score, mean (SD)	10.61 (3.75)

Legend. M, mean; SD, standard deviation.

**Table 3:** Descriptive statistics of the items of the HLS-EU-Q16 (16-item European Health Literacy Survey Questionnaire).

Domain	Items	M (SD)	Do Not Know/ Refusal *n* (%)	Very Difficult *n* (%)	Fairly Difficult *n* (%)	Fairly Easy *n* (%)	Very Easy *n* (%)
	On a scale from very easy to very difficult, how easy would you say it is to:						
	(1) Find information on treatments of illnesses that concern you?	2.28 (0.98)	4 (7)	5 (8.8)	23 (40.4)	21 (36.8)	4 (7)
	(2) Find out where to get professional help when you are ill?	2.58 (0.91)	2 (3.5)	2 (3.5)	22 (38.6)	23 (40.4)	8 (14.4)
	(3) Understand what your doctor says to you?	3.00 (0.71)	0 (0)	2 (3.5)	8 (14)	35 (61.4)	12 (21.1)
Health care domain	(4) Understand your doctor’s or pharmacist’s instruction on how to take a prescribed medicine?	3.19 (0.67)	0 (0)	1 (1.8)	5 (8.8)	33 (57.9)	18 (31.6)
	(5) Judge when you may need to get a second opinion from another doctor?	2.12 (1.18)	10 (17.5)	3 (5.3)	17 (29.8)	24 (42.1)	3 (5.3)
	(6) Use information the doctor gives you to make decisions about your illness?	2.82 (0.97)	3 (5.3)	1 (1.8)	11 (19.3)	30 (52.6)	12 (21.1)
	(7) Follow instructions from your doctor or pharmacist?	3.25 (0.71)	1 (1.8)	0 (0)	3 (5.3)	33 (57.9)	20 (35.1)
	(8) Find information on how to manage mental health problems like stress or depression?	2.09 (1.12)	8 (14)	5 (8.8)	22 (38.6)	18 (31.6)	4 (7)
	(9) Understand health warnings about behavior such as smoking, low physical activity and drinking too much?	3.30 (1.13)	4 (7)	1 (1.8)	3 (5.3)	15 (26.3)	34 (59.6)
Disease prevention domain	(10) Understand why you need health screenings?	3.58 (0.80)	1 (1.8)	1 (1.8)	2 (3.5)	13 (22.8)	40 (70.2)
	(11) Judge if the information on health risks in the media is reliable?	2.21 (1.08)	6 (10.5)	7 (12.3)	16 (28.1)	25 (43.9)	3 (5.3)
	(12) Decide how you can protect yourself from illness based on information in the media?	2.28 (1.16)	6 (10.5)	9 (15.8)	10 (17.5)	27 (47.4)	5 (8.8)
	(13) Find out about activities that are good for your mental well-being?	2.60 (1.08)	5 (8.8)	2 (3.5)	13 (22.8)	28 (49.1)	9 (15.8)
	(14) Understand advice on health from family members or friends?	2.23 (1.17)	9 (15.8)	3 (5.3)	14 (24.6)	28 (49.1)	3 (5.3)
Disease promotion domain	(15) Understand information in the media on how to get healthier?	2.44 (1.21)	7 (12.3)	3 (5.3)	15 (26.3)	22 (38.6)	10 (17.5)
	(16) Judge which everyday behavior is related to your health?	3.02 (0.94)	2 (3.5)	1 (1.8)	9 (15.8)	27 (47.4)	18 (31.6)

## Data Availability

The data sets included in the present study are available from the corresponding author upon reasonable request.

## Public Involvement Statement

There was no public involvement with any aspect of this research.

## Guidelines and Standards Statement

This manuscript was drafted against the STROBE Statement—Checklist of cohort studies.
